# Growth Arrest and DNA Damage Protein 45A Promotes PPRV Replication via the Downregulation of TBK1 Expression to Inhibit IFN‐β Signaling Pathway

**DOI:** 10.1096/fj.202500364R

**Published:** 2025-06-23

**Authors:** Haiyan Ding, Wenping Yang, Jinyan Wu, Junhuang Wu, Mengyi Wang, Jijun He, Ligang Yuan, Haixue Zheng, Youjun Shang, Dan Li

**Affiliations:** ^1^ State Key Laboratory for Animal Disease Control and Prevention, College of Veterinary Medicine, Lanzhou Veterinary Research Institute, Chinese Academy of Agricultural Sciences Lanzhou University Lanzhou China; ^2^ Gansu Province Research Center for Basic Disciplines of Pathogen Biology Lanzhou China; ^3^ College of Veterinary Medicine Gansu Agriculture University Lanzhou China; ^4^ Gansu Key Laboratory of Animal Generational Physiology and Reproductive Regulation Lanzhou China; ^5^ College of Life Science and Technology Gansu Agriculture University Lanzhou China

**Keywords:** GADD45A, interferon‐β, peste des petits ruminants virus, TANK‐binding kinase 1, V protein

## Abstract

Peste des petits ruminants virus (PPRV) is a highly contagious pathogen that severely impacts goats and sheep due to its high contagiousness and pathogenicity. Viruses rely on host proteins for their pathogenicity and replication, but the specific mechanisms facilitating PPRV replication by host proteins remain poorly understood. In this study, we identified goat growth arrest and DNA damage protein 45A (GADD45A) as a positive regulator of PPRV replication. Overexpression of GADD45A enhances PPRV replication, while its knockdown significantly inhibits PPRV replication. Furthermore, GADD45A suppresses SeV‐ or Poly(I:C)‐induced IFN‐β promoter and ISRE activation in a dose‐dependent manner, as well as the transcription of interferon‐stimulated genes (ISGs). We also demonstrate that goat GADD45A interacts with TANK‐binding kinase 1 (TBK1), leading to the downregulation of TBK1 expression. Co‐immunoprecipitation and confocal microscopy confirmed that GADD45A interacts with the PPRV V protein. Both GADD45A and V synergistically inhibit IFN‐β promoter activation and TBK1 expression, thereby promoting PPRV replication. Our findings suggest that GADD45A promotes PPRV replication by downregulating TBK1, offering new insights into host proteins that counteract innate immune responses during PPRV infection. These findings offer valuable insights into the role of host proteins in viral replication and immune evasion, shedding new light on how PPRV antagonizes innate immunity.

## Graphical abstract

A working model of host protein GADD45A regulation of PPRV replication. During PPRV infection, the viral protein V forms a complex with GADD45A in the cytoplasm. GADD45A interacts with TBK1 and inhibits its expression. Similarly, V also interacts with TBK1 and inhibits TBK1 expression. The synergistic inhibition of TBK1 by both GADD45A and V leads to the downregulation of IFN‐β promoter activation, ultimately promotes PPRV replication.

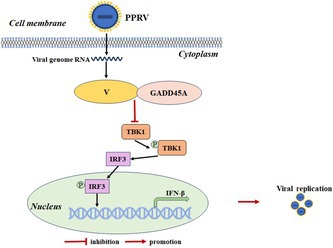

## 1 Introduction

Peste des Petits Ruminants Virus (PPRV) is a member of the Morbillivirus genus within the Paramyxoviridae family [1]. This virus primarily affects small ruminants, including goats and sheep. Infected animals typically exhibit symptoms such as fever, stomatitis, diarrhea, and pneumonia, which can lead to death in severe cases. PPRV poses a significant threat to the health of small ruminants and the sustainability of livestock industries [2]. PPRV is a single‐stranded negative‐sense RNA virus, approximately 15 948 nucleotides long, and encodes six structural proteins (N, P, M, F, H, L) and two non‐structural proteins (C and V) [3].

During viral infection, a variety of host proteins play critical roles in multiple stages of the virus entry, replication, and immune evasion. These host factors can either facilitate viral propagation or act as part of the host defense machinery to restrict viral replication through innate and adaptive immune responses. To ensure their survival and efficient replication, viruses have evolved strategies to hijack essential cellular pathways, such as transcription, translation, apoptosis, and immune signaling [4, 5, 6]. Research has demonstrated that vimentin recruits the E3 ubiquitin ligase NEDD4L, promoting ubiquitination and subsequent degradation of the viral nucleocapsid (N) protein, thereby inhibiting PPRV replication [7]. Additionally, plasminogen activator urokinase (PLAU) suppresses PPRV replication by upregulating VISA expression and reducing the fusion (F) protein‐mediated degradation of VISA [3]. On the other hand, the V protein contributes to viral immune evasion by directly interacting with key components of host signaling pathways. It binds to the Rel homology domain (RHD) of the NF‐κB p65 subunit, thereby suppressing NF‐κB transcriptional activity and downstream gene expression [8]. Notably, V protein expression significantly inhibits TNF‐α‐induced nuclear translocation of NF‐κB p65, further attenuating pro‐inflammatory signaling [8]. In addition, the V protein directly binds to signal transducer and activator of transcription 1 (STAT1) and STAT2, impairing interferon (IFN) mediated antiviral signaling and host immune responses [9]. Moreover, previous study has demonstrated that the V protein co‐localizes with mitochondrial antiviral signaling protein (MAVS), suggesting a potential role in interfering with MAVS‐dependent activation of innate immune [10]. Collectively, these findings highlight the central role of the V protein in modulating host immune responses, primarily through its interactions with key regulators of the NF‐κB and IFN signaling pathways. Although several host proteins involved in PPRV infection have been identified, further elucidation of virus–host interaction mechanisms is crucial for understanding viral pathogenicity and developing effective control strategies.

The GADD45A gene encodes a protein of approximately 18 kDa that plays several critical roles in cellular functions, including cell cycle regulation, DNA damage repair, apoptosis, stress response, and immune modulation. This protein is localized in both the cell nucleus and cytoplasm [11]. GADD45A is essential for cellular stress responses and the maintenance of genomic stability [12, 13]. Research has demonstrated that the HCV NS5A protein can directly interact with GADD45A, inhibiting its mRNA transcription and protein expression, while also enhancing the uptake of free fatty acids (FFAs), thereby promoting viral replication [14, 15]. Notably, GADD45A protein expression is elevated in nasopharyngeal carcinoma tissues compared to non‐tumor tissues [16]. Additionally, studies have shown that Aristolochic acids (AA) can inhibit ferroptosis through the p53/GADD45A/NRF2/SLC7A11 axis, promoting the growth of hepatocellular carcinoma [17]. GADD45A is upregulated in liver cirrhosis (LC), hepatocellular carcinoma (HC), acute hepatic failure (AHF), and non‐alcoholic fatty liver disease (NAFLD). It regulates various physiological processes through multiple signaling pathways, including P38 MAPK, NF‐κB, mTOR/STAT3, P21, PCNA, and PI3K/Akt [18].

Pattern recognition receptors (PRRs) can recognize pathogen‐associated molecular patterns (PAMPs) [19]. The activation of PRRs triggers the expression of pro‐inflammatory cytokines, the recruitment of immune cells, and the production of type I and type III interferons, which subsequently induce interferon‐stimulated genes (ISGs) and establish an “antiviral” state [20]. However, for viruses to successfully infect host cells and replicate, they must evade the host's innate immune mechanisms. The PPRV V protein inhibits the activation of interferon regulatory factor 3 (IRF3), preventing the production of IFN‐β; besides, the V protein interferes with the phosphorylation of STAT1, inhibiting the downstream effects of the interferon signaling pathway [21]. The P protein competes with TANK‐binding kinase 1 (TBK1) for binding to the IRF association domain (IAD) of IRF3, leading to reduced phosphorylation of IRF3. This competition prevents the dimerization and nuclear translocation of IRF3, ultimately inhibiting the production of type I interferons and promoting viral replication [22]. TBK1, a member of the mitogen‐activated protein kinase (MAPK) family, is a pivotal protein involved in multiple signaling pathways and primarily functions in innate immune responses. Both host and viral factors can target TBK1 [23], creating a dynamic balance of mutual antagonism between the host and the virus. TBK1 mediates the activation of IRF3, which induces the production of type I interferons (IFN‐α/β) following viral infection [24, 25].

In our study, we observed that the transcription and protein level of GADD45A were elevated in PPRV‐infected Goat Alveolar Macrophages (GAM) cells. GADD45A not only promotes PPRV replication but also activates the TBK1‐triggered RLR signaling pathway. Furthermore, GADD45A interacts with the PPRV V protein, which may synergistically suppress TBK1 expression. These findings unveil new mechanisms through which host proteins contribute to the innate immune responses induced by PPRV infection.

## 2 Materials and Methods

### 2.1 Cell Culture and Virus Infection

GAM cells were isolated from healthy 6‐week‐old goats that had passed quarantine inspection. GAM cells were cultured in RPMI 1640 medium containing 10% fetal bovine serum (FBS) (Gibco) and 1% penicillin–streptomycin. HEK293T cells were cultured in DMEM medium containing 10% FBS and 1% penicillin–streptomycin–gentamicin. Vero cells were cultured in M199 medium supplemented with 2% FBS and 1% penicillin–streptomycin–gentamicin. All cells were maintained in a humidified incubator at 37°C with 5% CO_2_. The attenuated strain of PPRV Nigeria 75/1 was obtained in our laboratory using Vero cells. The viral titer was assessed using cytopathic effect (CPE) and quantified by 50% tissue culture infectious dose (TCID_50_). The multiplicity of infection (MOI) was determined based on the viral titers for each cell line. For viral infection, Vero/GAM cells were seeded at a density of 1 × 10^6^ cells/mL in 12‐well cell culture plates and either infected with or uninfected by PPRV (Nigeria 75/1). After a 2‐h adsorption period, the infected GAM cells were maintained in RPMI 1640 medium containing 2% FBS, and the infected Vero cells were maintained in M199 medium containing 1% FBS.

### 2.2 Reagents and Antibodies

GAPDH antibody was purchased from Abcam. Monoclonal anti‐HA, anti‐Myc, and anti‐Flag mouse and rabbit antibodies were purchased from Sigma‐Aldrich (USA). Polyclonal rabbit anti‐IRF3, anti‐p‐IRF3, anti‐TBK1, anti‐p‐TBK1, anti‐IκBα, anti‐p‐IκBα, anti‐P65, and anti‐p‐P65 antibodies were purchased from Cell Signaling Technology (USA). Anti‐mouse IgG and anti‐rabbit IgG antibodies were purchased from Thermo Scientific. Polyclonal mouse GADD45A antibody was purchased from Novus Biologicals (USA); polyclonal rabbit GADD45A antibody was purchased from Proteintech (Wuhan). CoraLite594‐conjugated goat anti‐mouse IgG (H + L) and fluorescein isothiocyanate (FITC)‐conjugated goat anti‐rabbit IgG (H + L) antibodies were purchased from Cell Signaling Technology (CST).

### 2.3 Transfection and Luciferase Assay

HEK293T cells were evenly seeded in 48‐well plates and allowed to grow to 60%–70% confluence. Transfection was performed using the standard calcium phosphate precipitation method. Briefly, cells were co‐transfected with a plasmid expressing the gene of interest, a firefly luciferase reporter construct driven by either the IFN‐β or ISRE promoter, and the Renilla luciferase‐expressing control plasmid pRL‐TK. Following 24 h of post‐transfection incubation, luciferase activity was measured using the Dual‐Luciferase Reporter Assay System (Promega), according to the manufacturer's protocol. Firefly luciferase signals were normalized to the corresponding Renilla luciferase values to account for variations in transfection efficiency. The results are expressed as the fold change in luciferase activity relative to the control group. Each experiment was repeated at least three times.

### 2.4 siRNA Knockdown

The siRNA targeting the GADD45A sequence was synthesized by Sangon Biotech (China). The GADD45A siRNA target sequence is as follows: Forward (F): 5′‐GCGAGAAUGAUAUCGACAUTT‐3′, Reverse (R): 5′‐AUGUCGAUAUCAUUCUCGCTT‐3′. The Control‐RNAi and GADD45A‐RNAi were co‐transfected into GAM cells using the jetPRIME reagent (Polyplus‐transfection) for 48 h.

### 2.5 Real‐Time Quantitative Reverse Transcription PCR

Total RNA was extracted using TRIzol reagent (Sigma, USA) and quantified using a NanoDrop ND‐2000C spectrophotometer. cDNA was synthesized from the purified total RNA by using PrimeScript RT Master Mix (Perfect Real Time) (Takara, Beijing). The cDNA of target gene transcripts was quantified using the SYBR Green method. The total reaction volume was 25 μL, consisting of 12.5 μL of 2 × SYBR Premix Ex Taq II, 0.5 μL (10 μM) forward primer, 0.5 μL (10 μM) reverse primer, 8.5 μL H_2_O, and 3 μL cDNA product. The PCR cycling conditions were as follows: initial denaturation at 95°C for 10 min, followed by 40 cycles of 95°C for 15 s and 60°C for 1 min. GAPDH was used as the housekeeping gene. The relative expression levels of the target genes were calculated using the 2^−ΔΔCT^ method and expressed as fold changes. The primers for RT‐PCR are listed in Table 1.

**TABLE 1:** Primers used in this study.

Primers	Sequences (5′–3′)
Human IFNB	F:TTGTTGAGAACCTCCTGGCT R:TGACTATGGTCCAGGCACAG
Human IP10	F:GGTGAGAAGAGATGTCTGAATCC R:GTCCATCCTTGGAAGCACTGCA
Human ISG56	F:TCATCAGGTCAAGGATAGTC R:CCACACTGTATTTGGTGTCTAGG
Human RANTES	F:GGCAGCCCTCGCTGTCATCC R:GCAGCAGGGTGTGGTGTCCG
Human GAPDH	F:GAGTCAACGGATTTGGTCGT R:GACAAGCTTCCCGTTCTCAG
Goat GADD45A	F: CAGGCATTCTGCTGCGA R: GGAGCAAGAGTTCGGCCA
Goat GAPDH	F:CACTGCCACCCAGAAGACT R:CAGATCCACAACGGACACG

### 2.6 Co‐Immunoprecipitation (Co‐IP) Assay and Immunoblotting Analysis

For transient transfections and Co‐IP experiments, cells were harvested at 24 h after transfection with the indicated plasmids. Cells were washed once with PBS and then lysed in 1 mL of freshly prepared lysis buffer (20 mM Tris–HCl, pH 7.5, 150 mM NaCl, 1% Triton X‐100, 1 mM EDTA, 10 μg/mL aprotinin, 10 μg/mL leupeptin, and 1 mM phenylmethylsulfonyl fluoride) for 20 min at 4°C. For each immunoprecipitation, 400 μL of the lysate was incubated with 1 μL of the indicated antibody or control IgG and 40 μL of 1:1 Gammabind G Sepharose beads (Amersham Biosciences) for 4 h. The agarose beads were washed three times with 1 mL of lysis buffer containing 0.5 M NaCl. Samples were subjected to immunoblotting, and proteins were separated by electrophoresis on a 12% SDS‐PAGE gel and transferred to nitrocellulose membranes (Pall Corporation, Port Washington, NY, USA). The membrane was blocked with 5% non‐fat milk in Tris‐buffered saline with Tween 20 (TBST) for 30 min at room temperature. The membrane was incubated with the specified primary antibody overnight at 4°C, then washed three times with TBST, and incubated with horseradish peroxidase‐conjugated secondary antibody (1:5000) for 1 h at 37°C. For endogenous experiments, GAMs or Vero cells were infected with PPRV for designated times. Co‐IP and immunoblotting were performed as described above.

To assess the phosphorylation status of target proteins, equal amounts of protein were separated by SDS‐PAGE and transferred onto NC membranes. The membranes were incubated with primary antibodies specific for the phosphorylated forms of the target proteins (p‐IRF3, p‐TBK1, p‐IκBα, or p‐p65) overnight at 4°C. Following incubation with horseradish peroxidase (HRP)‐conjugated secondary antibodies, immunoreactive bands were visualized using enhanced chemiluminescence (ECL) reagents.

### 2.7 Confocal Microscopy

Human embryonic kidney 293T (HEK293T) cells (2 × 10^5^) were uniformly seeded in 35‐mm confocal glass‐bottom dishes for exogenous confocal imaging. Vero cells (2 × 10^5^) were infected with PPRV (MOI = 1) for 24 h. Cells were fixed overnight at 4°C with 1 mL of 4% paraformaldehyde. Cells were washed three times with PBS, permeabilized with PBS containing 1% Triton X‐100 for 5 min at room temperature, and washed three times with PBS. Cells were blocked with PBS containing 5% bovine serum albumin (BSA) for 30 min at room temperature. Cells were incubated with specific primary antibodies (1:300) overnight at 4°C. After washing three times with PBS, cells were incubated with specific secondary antibodies (1:500) and stained with 4′,6‐diamidino‐2‐phenylindole (DAPI) for 5 min. Finally, cells were observed under a confocal laser microscope (Leica, Germany).

### 2.8 Statistical Analysis

Data are presented as mean ± standard deviation (SD) from at least three independent experiments. Data graphs were created using GraphPad Prism 8.0. Data were analyzed using one‐way or two‐way ANOVA with Duncan's post‐test utilizing SPSS 16.0. *p* values < 0.05 were considered statistically significant. All data have been included within the article.

## 3 Results

### 3.1 Promotion of PPRV Replication by Goat GADD45A

**FIGURE 1:**
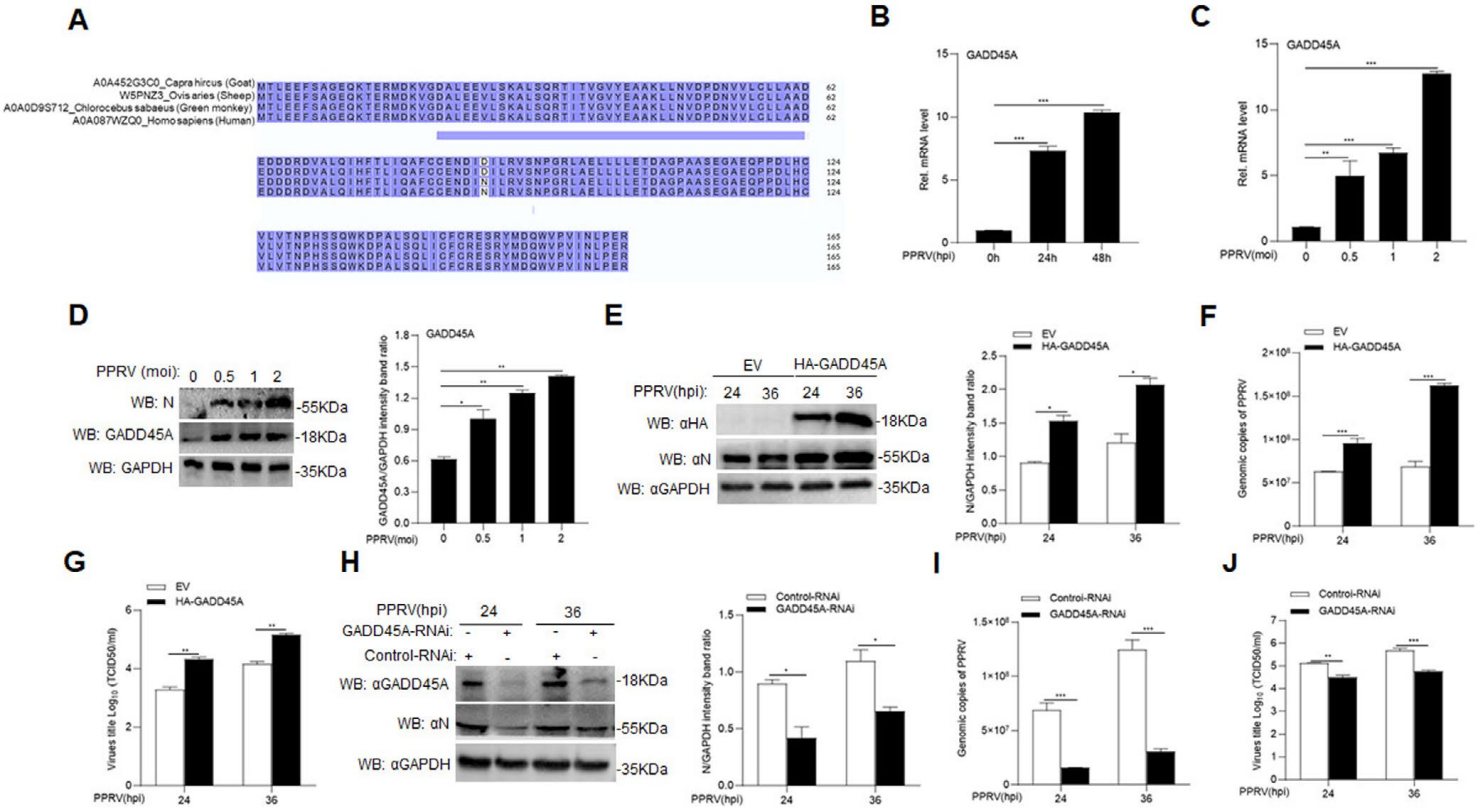
Goat GADD45A promotes the replication of PPRV. (A) Sequence comparison of GADD45A among selected mammalian species: Goats, sheep, green monkeys, and humans. (B) GAM cells (2 × 10^6^) were infected with PPRV (MOI = 1.0) at 0, 24, and 48 h, and the levels of goat GADD45A mRNA were measured. (C) GAM cells (2 × 10^6^) were infected with different doses of PPRV (MOI = 0, 0.5, 1, 2), and the levels of GADD45A mRNA were measured. (D) GAM (2 × 10^6^) cells were infected with PPRV (MOI = 0, 0.5, 1, 2), and samples were analyzed by Western blot (WB) using specific antibodies. Quantification of GADD45A protein levels normalized to GAPDH by densitometry. (E–G) Vero cells (5 × 10^5^) were transfected with 1.5 μg of HA‐GADD45A plasmid or empty vector (EV) for 24 h. Subsequently, the cells were infected with PPRV (MOI = 1.0) for 24 and 36 h, and the levels of PPRV N protein, quantification of N protein levels normalized to GAPDH by densitometry (E), genome copy number (F), and viral titer (G) were measured. (H–J) GAM cells (2 × 10^6^) were transfected with GADD45A‐RNAi or Control‐RNAi for 48 h. Then, the cells were infected with PPRV (MOI = 1.0) for 24 h. The levels of PPRV N protein, quantification of N protein levels normalized to GAPDH by densitometry (H), genome copy number (I), and viral titer (J) were measured. Data are presented as the mean ± standard deviation (SD) (*n* = 3). *, *p* < 0.05; **, *p* < 0.01; ***, *p* < 0.001. MOI, multiplicity of infection; RNAi, interfering RNA.

The X protein (HBx) of hepatitis B virus (HBV) has been shown to upregulate the expression of GADD45A [26]. The regulatory protein Tax, encoded by human T‐cell leukemia virus type 1 (HTLV‐1), is considered a viral oncoprotein and has been demonstrated to induce the upregulation of GADD45A expression in host cells [27]. We conducted a bioinformatics analysis of the GADD45A gene in goats (GenBank accession: XM_005678467.3). Our results indicate that the amino acid sequence of GADD45A is 100% identical between goat ( *Capra hircus* ) and sheep ( *Ovis aries* ), and exhibits over 99% identity among goat, human ( *Homo sapiens* ), and green monkey ( *Chlorocebus sabaeus* ). Additionally, domain analysis revealed that all key functional domains are conserved across these species (Figure 1A). This high degree of sequence conservation, coupled with the preservation of critical domains, suggests a conserved functional role of GADD45A across species and supports the biological relevance of using goat‐derived GADD45A in our experimental system. Upon infection of well‐maintained and contamination‐free GAM cells with the PPRV (Nigeria 75/1 strain), we observed a time‐ and dose‐dependent upregulation of GADD45A mRNA expression (Figure 1B,C; *p* < 0.01). This transcriptional increase was paralleled by a corresponding elevation in GADD45A protein levels (Figure 1D; *p* < 0.05). These findings prompted us to investigate the role of GADD45A in PPRV infection. To assess the impact of GADD45A overexpression on PPRV replication, Vero cells were transfected with either an empty vector (EV) or HA‐GADD45A, followed by PPRV infection. The results showed that, compared to EV‐transfected cells, overexpression of HA‐GADD45A resulted in higher levels of PPRV N protein expression (Figure 1E; *p* < 0.05), an increase in PPRV genome copies (Figure 1F; *p* < 0.01), and elevated viral titers (Figure 1G; *p* < 0.01). To explore the role of endogenous GADD45A in PPRV replication, we used specific siRNAs to knock down GADD45A in GAM cells. After transfection with either GADD45A‐RNAi or Control‐RNAi and subsequent PPRV infection, we found that GADD45A knockdown led to reduced levels of PPRV N protein (Figure 1H; *p* < 0.05), lower genome copy numbers (Figure 1I; *p* < 0.01), and decreased viral titers (Figure 1J; *p* < 0.01).

### 3.2 Negative Regulation of the IFN Signaling Pathway by GADD45A

**FIGURE 2:**
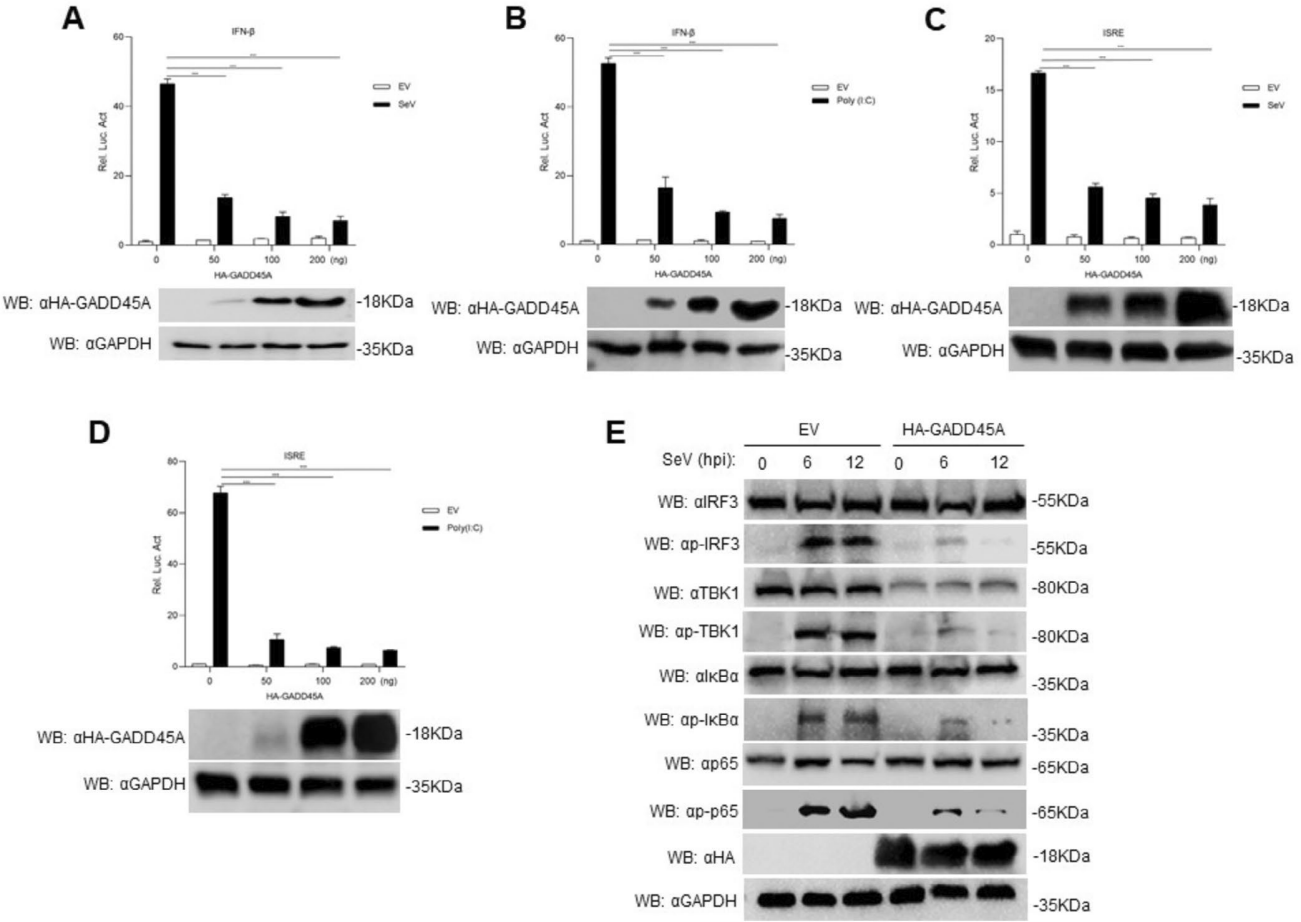
GADD45A negatively regulates the IFN‐β signaling pathway. (A–D) HEK293T cells (1 × 10^5^) were transfected with 0.1 μg of the IFN‐β reporter gene or 0.1 μg of ISRE, 10 ng of TK, and different amounts of HA‐GADD45A plasmid (0, 50, 100, and 200 ng) for 24 h. Then, the cells were either infected with Sendai virus (SeV) (MOI = 1.0) for 12 h or transfected with Poly(I:C) (4 μg/mL) for 18 h, and luciferase activity was measured. (E) HEK293T cells (2 × 10^5^) were transfected with 1.5 μg of HA‐GADD45A plasmid for 24 h, followed by infection with SeV for 6 and 12 h. Protein extracts were analyzed by Western blot using specific antibodies, with GAPDH serving as a loading control. Data are presented as the mean ± SD (*n* = 3). *, *p* < 0.05; **, *p* < 0.01; ***, *p* < 0.001. IFN, interferon; ISRE, Interferon‐Stimulated Response Element; TK, Tyrosine Kinase.

To investigate whether GADD45A antagonizes IFN expression, we performed dose‐dependent transfections of HEK293T cells using either the IFN‐β promoter reporter plasmid or an ISRE‐containing reporter plasmid, together with the internal control plasmid pRL‐TK, and either the HA‐GADD45 expression plasmid or EV plasmid, all at equivalent concentrations. Following transfection, cells were either stimulated with Sendai virus (SeV) or transfected with the RNA analog Poly(I:C). The IFN‐β transcription level was assessed using a dual‐luciferase reporter system assay. Results showed that GADD45A significantly inhibited the activation of the IFN‐β promoter in a dose‐dependent manner, both in response to Sendai virus (SeV) (Figure 2A; *p* < 0.001) and Poly(I:C) stimulation (Figure 2B; *p* < 0.001). Additionally, GADD45A dose‐dependently inhibited ISRE activation induced by SeV (Figure 2C; *p* < 0.001) or Poly(I:C) (Figure 2D; *p* < 0.001). RIG‐I‐like receptors (RLRs) play a crucial role in the host's defense against RNA virus infections. Overexpression of GADD45A not only significantly suppresses TBK1 expression, but also reduces the phosphorylation levels of key signaling molecules, including p‐IRF3, p‐TBK1, p‐IκBα, and p‐p65 (Figure 2E). Phosphorylation is mostly inhibited after 12 h and not so much after 6 h SeV stimulation. This delayed inhibitory effect may be attributed to the accumulation of GADD45A protein or its interactions with other cellular factors over time. These findings suggest that while the RLR‐mediated signaling pathway is initially activated, its functions are progressively suppressed by GADD45A, resulting in reduced phosphorylation at later time points.

### 3.3 GADD45A Inhibits the Transcription of IFN Downstream Factors Associated With Interferon Signaling

**FIGURE 3:**
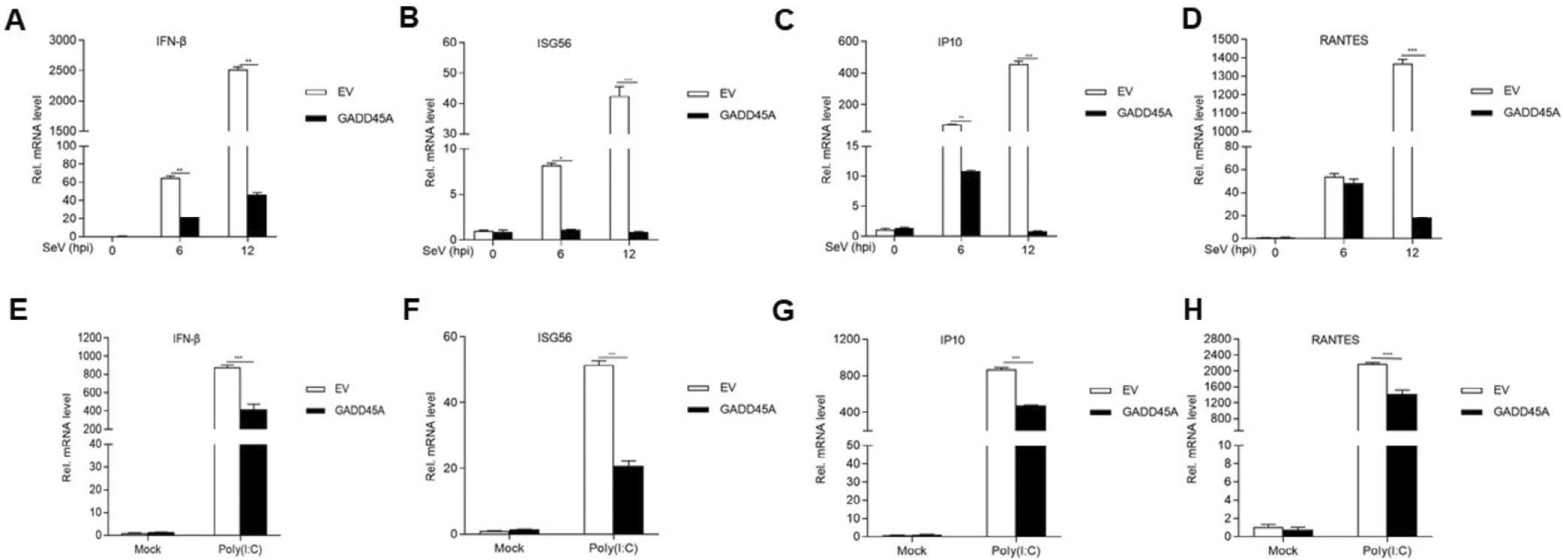
GADD45A inhibits the transcription of IFN downstream factors associated with interferon signaling. (A–D) HEK293T cells (3 × 10^5^) were transfected with 1.5 μg of HA‐GADD45A or 1.5 μg of EV plasmid for 24 h. The cells were infected with SeV (MOI = 1.0) for 6 or 12 h. Subsequently, the transcription levels of downstream antiviral genes were measured by RT‐PCR. (E–H) HEK293T cells (3 × 10^5^) were transfected with 1.5 μg of HA‐GADD45A or 1.5 μg of EV in 12‐well plates for 24 h. The cells were transfected with Poly(I:C) (4 μg/mL) for 18 h. Subsequently, the transcription levels of IFN‐β, ISG56, IP10, and RANTES genes were measured by RT‐PCR. Data are presented as the mean ± SD (*n* = 3). *, *p* < 0.05; **, *p* < 0.01; ***, *p* < 0.001. ISG, Interferon‐Stimulated Gene; IP10, Interferon gamma‐induced protein 10; RANTES, regulated on activation, normal T cell expressed and secreted.

To further investigate the role of GADD45A on the transcription of downstream factors associated with interferon signaling, HEK293T cells were transfected with a GADD45A expression plasmid and then infected with SeV or transfected with Poly(I:C). We observed that the transcription levels of several genes, including IFN‐β, ISG56, IP10, and RANTES, were significantly reduced in GADD45A‐overexpressing HEK293T cells compared to control cells by real‐time PCR (RT‐qPCR) assay (Figure 3A–D; *p* < 0.05). Furthermore, in GADD45A‐overexpressing HEK293T cells transfected with Poly(I:C), we observed a similar decrease in the transcription levels of IFN‐β, ISG56, IP10, and RANTES relative to control cells (Figure 3E–H; *p* < 0.001). These results indicate that GADD45A negatively regulates the RLR‐mediated signaling pathway.

### 3.4 GADD45A Interacts With TBK1

**FIGURE 4:**
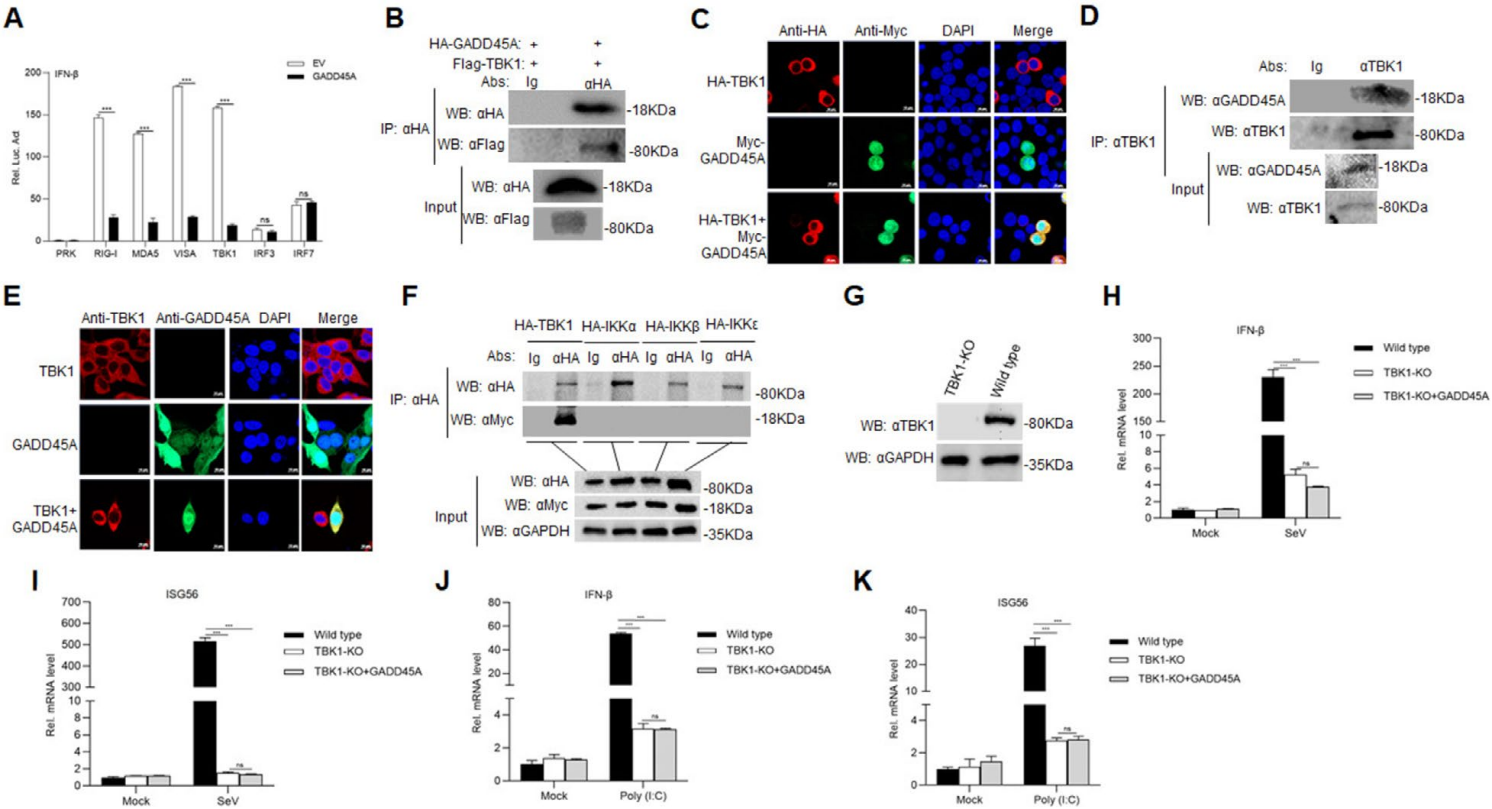
GADD45A interacts with TBK1. (A) HEK293T cells (1 × 10^5^) were transfected with 0.1 μg of IFN‐β promoter plasmid, 10 ng of TK, expression plasmids of GADD45A (0.1 μg), and the other indicated plasmids (each 0.1 μg) in 48‐well plates. Luciferase activity was measured 24 h post‐transfection. (B) HEK293T cells (2 × 10^6^) in 10‐cm cell culture dishes were co‐transfected with 10 μg of HA‐GADD45A and 10 μg of Flag‐TBK1. After 24 h, the cells were processed for Co‐IP using an anti‐HA antibody, followed by Western blot analysis. (C) HEK293T cells (2 × 10^5^) in 35 mm dishes were transfected with 1 μg of Myc‐GADD45A or 1 μg of HA‐TBK1 individually, or co‐transfected with 1 μg of Myc‐GADD45A and 1 μg of HA‐TBK1. After 24 h, the cells were fixed overnight at 4°C with 4% paraformaldehyde and stained with mouse anti‐Myc and rabbit anti‐HA antibodies for indirect immunofluorescence detection of Myc‐GADD45A (green) and HA‐TBK1 (red). Nuclei were stained with DAPI (blue). (D) GAM cells (2 × 10^7^) were harvested for Co‐IP by an anti‐GADD45A antibody, followed by Western blot analysis. (E) Vero cells were seeded in 35 mm confocal dishes and grown to 60%–80% confluence. The cells were fixed overnight at 4°C with 4% paraformaldehyde and stained with rabbit anti‐TBK1 (green) and mouse anti‐GADD45A (red) antibodies for indirect immunofluorescence. Nuclei were stained with DAPI (blue). (F) HEK293T cells (2 × 10^6^) were seeded in 10 cm culture dishes and transfected with 10 μg of Myc‐GADD45A plasmid along with either 10 μg of HA‐TBK1, HA‐IKKα, HA‐IKKβ, or HA‐IKKε expression plasmids. After 24 h, cells were harvested and subjected to Co‐IP using an anti‐HA antibody, followed by Western blot analysis. (G–K) TBK1‐knockout cells (3 × 10^5^) were transfected with 0.5 μg of GADD45A plasmid and then infected with SeV for 12 h or transfected with Poly(I:C) for 18 h. RT‐qPCR was used to detect the mRNA levels of IFN‐β, ISG56. Data are presented as the mean ± SD (*n* = 3). ***, *p* < 0.001.

To identify potential molecular targets in the pathways influenced by GADD45A, we examined its effect on the activation of the IFN‐β promoter mediated by RLR signaling molecules. HEK293T cells were co‐transfected with HA‐GADD45A and key components of the RLR pathway, including HA‐RIG‐I, HA‐MDA5, HA‐VISA, HA‐TBK1, HA‐IRF3, and HA‐IRF7, along with the pIFN‐β‐Luc plasmid. The results showed that overexpression of GADD45A inhibited IFN‐β promoter activation induced by RIG‐I, MDA5, VISA, and TBK1, but had no effect on the activation mediated by IRF3 and IRF7 (Figure 4A). Furthermore, we cotransfected the HEK293T cells with GADD45A and TBK1 expression plasmids. Co‐IP and confocal microscopy analyses demonstrated an interaction between GADD45A and TBK1 in the exogenous system (Figure 4B). Confocal microscopy further revealed that GADD45A and TBK1 were colocalized in the cytoplasm (Figure 4C). Endogenous Co‐IP experiments also confirmed the interaction between GADD45A and TBK1 (Figure 4D), with confocal microscopy again showing their co‐localization in the cytoplasm in PPRV‐infected cells (Figure 4E). To further clarify the specificity of GADD45A for TBK1, we performed Co‐IP experiments using HEK293T cells transfected with Myc‐tagged GADD45A and various HA‐tagged kinases, including TBK1, IKKα, IKKβ, and IKKε. The results showed that GADD45A specifically interacts with TBK1, but not with other IKK family members (Figure 4F). To explore the role of TBK1 in cellular signaling pathways and antiviral immune responses, we utilized TBK1 knockout (KO) cell lines alongside control cells, both of which were transfected with HA‐GADD45A (Figure 4G). Following stimulation with SeV or Poly(I:C), we analyzed the mRNA levels of IFN‐β and ISG56 using RT‐qPCR. The results demonstrated that both the TBK1‐KO cell line and the TBK1‐KO cell line transfected with HA‐GADD45A exhibited significantly reduced mRNA levels of IFN and ISG56 after treatment with SeV or Poly(I:C) compared to the wild‐type control cells (Figure 4H–K; *p* < 0.001). Following viral stimulation, under normal cellular conditions, the interaction between GADD45A and TBK1 suggests that GADD45A may inhibit the activity of this pathway through a negative feedback mechanism by interfering with TBK1 activation.

### 3.5 GADD45A Inhibits the Expression of TBK1

**FIGURE 5:**
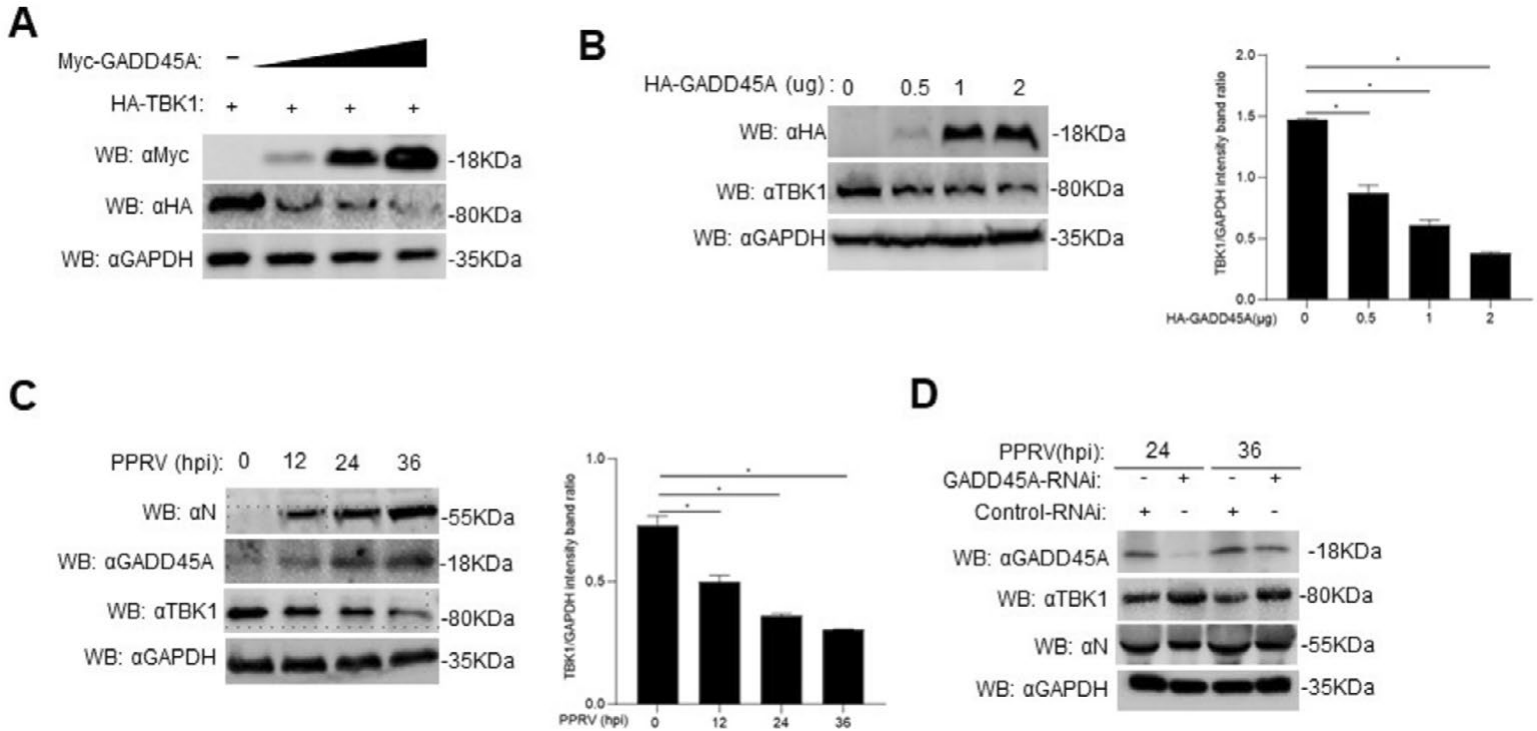
GADD45A inhibits the expression of TBK1. (A) HEK293T cells (2 × 10^5^) were transfected with 0.5 μg of HA‐TBK1 and varying amounts of Myc‐GADD45A (0, 0.5, 1, and 2 μg) for 24 h, followed by Western blot analysis. (B) Vero cells (4 × 10^5^) were transfected with different amounts of HA‐GADD45A (0, 0.5, 1, and 2 μg) for 24 h. Subsequently, Western blot analysis was performed and TBK1 protein levels were quantified by densitometry and normalized to GAPDH. (C) GAM cells (2 × 10^6^) were infected with PPRV (MOI = 1.0) at 0, 12, 24, and 36 h. Specific antibodies were used for Western blot analysis. Quantification of TBK1 protein levels was normalized to GAPDH by densitometry. (D) GAM cells (2 × 10^6^) were transfected with GADD45A‐RNAi or Control‐RNAi for 48 h. Then, the cells were infected with PPRV (MOI = 1.0) for 24 or 36 h, and specific antibodies were used for Western blot analysis.

To further elucidate the impact of GADD45A on TBK1 expression, we cotransfected with HA‐TBK1 and Myc‐GADD45A in HEK293T cells, and the results showed that GADD45A inhibited the expression of TBK1in a dose‐dependent manner (Figure 5A). Moreover, overexpression of GADD45A in Vero cells resulted in decreased endogenous TBK1 expression (Figure 5B; *p* < 0.05). Upon PPRV infection of GAM cells, we observed a time‐dependent increase in PPRV replication, accompanied by elevated GADD45A expression and reduced TBK1 levels (Figure 5C; *p* < 0.05). In PPRV‐infected GADD45A‐knockdown GAM cells, we noted a significant rise in TBK1 expression and a decrease in PPRV replication compared to the control group (Figure 5D).

### 3.6 GADD45A Interacts With PPRV V Protein and V Inhibits TBK1 Expression

**FIGURE 6:**
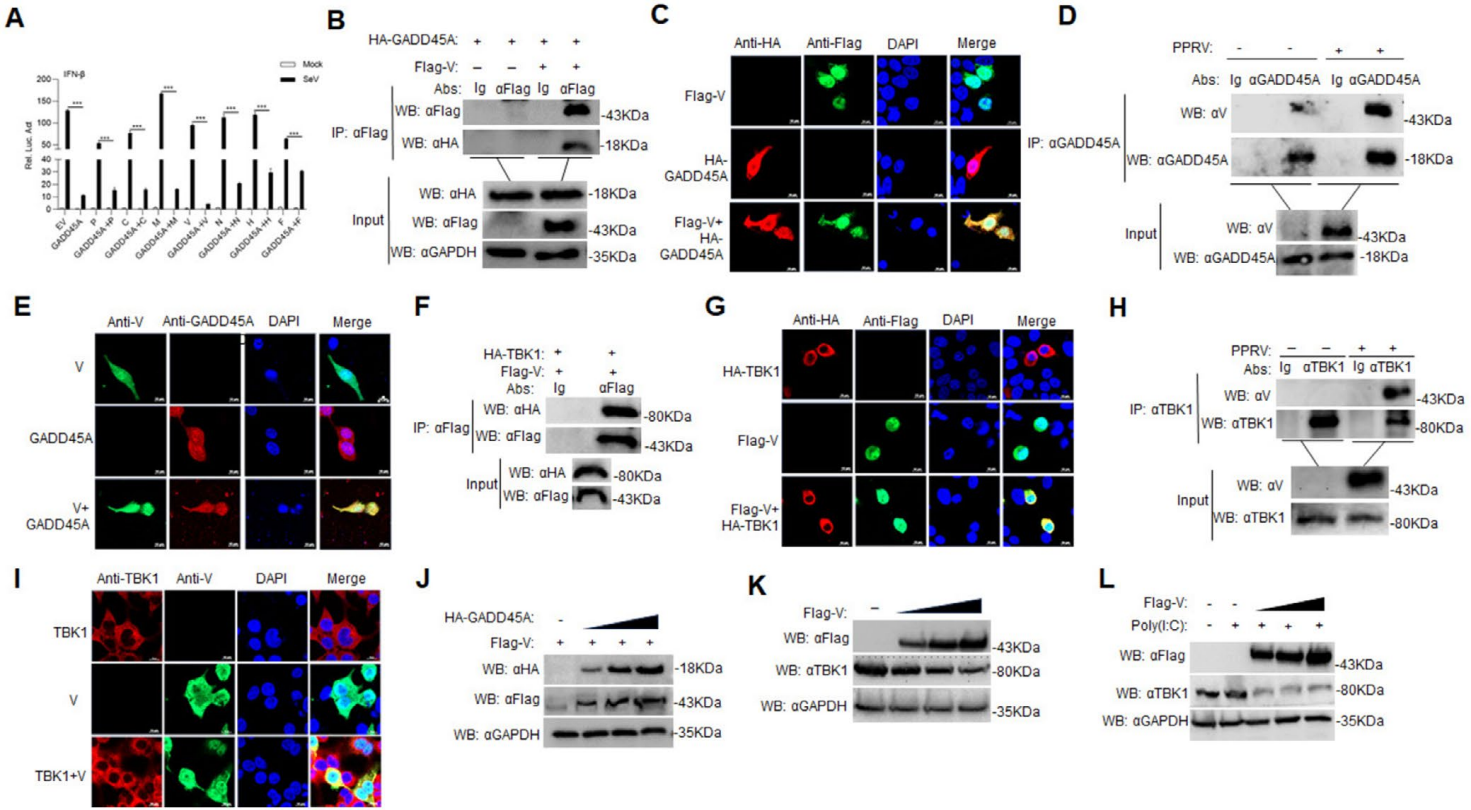
GADD45A interacts with PPRV V protein that inhibits TBK1 expression. (A) HEK293T cells (1 × 10^5^) were transfected with IFN‐β promoter (0.1 μg), TK (10 ng), expression plasmids for GADD45A (0.1 μg), and viral protein (each 0.1 μg) for 24 h, followed by SeV stimulation for 12 h. Luciferase activity was measured. (B) HEK293T cells were co‐transfected with HA‐GADD45A plasmid (10 μg), EV (10 μg) or Flag‐V (10 μg) in 10 cm cell culture dishes. Cells were collected and processed for Co‐IP using an anti‐Flag antibody, followed by Western blot analysis. (C) Flag‐V (1 μg) or Myc‐GADD45A (1 μg) were transfected individually, or Flag‐V (1 μg) and Myc‐GADD45A (1 μg) were co‐transfected into HEK293T cells (2 × 10^5^) for 24 h. Cells were fixed overnight at 4°C and stained with mouse anti‐Flag and rabbit anti‐Myc antibodies for indirect immunofluorescence detection of Flag‐V (green) and Myc‐GADD45A (red). DAPI (blue) staining was used to indicate nuclei. (D) GAM cells (2 × 10^7^) were infected or uninfected with PPRV (MoI = 5) for 24 h, and the cells were harvested. Co‐IP was performed using an anti‐GADD45A antibody, followed by Western blot analysis. (E) Vero cells (2 × 10^5^) were infected or uninfected with PPRV (MOI = 2.0) for 24 h, fixed overnight at 4°C, and stained with rabbit anti‐GADD45A and mouse anti‐V antibodies for indirect immunofluorescence detection of V (green) and GADD45A (red). Nuclei were indicated by DAPI (blue) staining. (F) Flag‐V (10 μg) and HA‐TBK1 (10 μg) were co‐transfected into HEK293T cells (2 × 10^6^) for 24 h. The cells were collected and processed for Co‐IP using an anti‐Flag antibody, followed by Western blot analysis. (G) Flag‐V (1 μg) or HA‐TBK1 (1 μg) were transfected individually, or Flag‐V (1 μg) and HA‐TBK1 (1 μg) were co‐transfected into HEK293T cells (2 × 10^5^) for 24 h. Cells were fixed overnight at 4°C and stained with mouse anti‐Flag and rabbit anti‐HA antibodies for indirect immunofluorescence detection of Flag‐V (green) and HA‐TBK1 (red). Nuclei were indicated by DAPI (blue) staining. (H) GAM cells (2 × 10^7^) were infected or uninfected with PPRV (MOI = 5) for 24 h, and the treated cells were harvested. Co‐IP was performed using an anti‐TBK1 antibody, followed by Western blot analysis. (I) Vero (2 × 10^5^) cells were infected or uninfected with PPRV (MOI = 1.0) for 24 h, fixed overnight at 4°C, and stained with rabbit anti‐TBK1 and mouse anti‐V antibodies for indirect immunofluorescence detection of V (green) and TBK1 (red). Nuclei were indicated by DAPI (blue) staining. (J) HEK293T cells were co‐transfected with Flag‐V (0.5 μg) and HA‐GADD45A (0, 0.5, 1, and 1.5 μg) for 24 h, followed by Western blot analysis. (K) HEK293T cells was transfected Flag‐V (0, 0.5, 1, 2 μg) in 12‐well plates for 24 h, followed by Western blot analysis. (L) HEK293T cells were co‐transfected Flag‐V (0, 0.5, 1, and 1.5 μg) and EV for 24 h, followed by transfection with Poly(I:C) for 18 h. All samples were collected for Western blot analysis. EV, empty vector. ***p < 0.001.

To further investigate the interaction between GADD45A and PPRV proteins, we transfected HEK293T cells with HA‐GADD45A either alone or in combination with various PPRV proteins (Flag‐P, Flag‐C, Flag‐M, Flag‐V, Flag‐N, Flag‐H, Flag‐F) along with the IFN‐β‐Luc plasmid, followed by infection with SeV. The results indicated that co‐transfection of GADD45A and the V protein resulted in the lowest level of IFN‐β promoter activation compared to other viral proteins (Figure 6A). Additionally, we performed Co‐IP assays by co‐transfecting HA‐GADD45A and Flag‐V into HEK293T cells, revealing an interaction between GADD45A and V (Figure 6B). To further analyze their co‐localization, we cotransfected the HEK293T cells with HA‐GADD45A and Flag‐V plasmids. Confocal microscopy confirmed that GADD45A and V co‐localized within the cells (Figure 6C). Endogenous Co‐IP experiments also validated the interaction between GADD45A and V in PPRV‐infected GAMs (Figure 6D). Moreover, in PPRV‐infected Vero cells, confocal microscopy demonstrated that GADD45A and V co‐localized as well (Figure 6E). We then explored the interaction between V and TBK1 by co‐transfecting HA‐TBK1 and Flag‐V plasmids into HEK293T cells, followed by Co‐IP and confocal microscopy analyses. The results confirmed that V interacts with TBK1 (Figure 6F), and confocal microscopy illustrated the co‐localization of exogenously expressed HA‐TBK1 and Flag‐V in HEK293T cells (Figure 6G). Endogenous Co‐IP experiments further showed that TBK1 interacts with V in PPRV‐infected GAMs (Figure 6H), and confocal microscopy also indicated co‐localization of V and TBK1 in PPRV‐infected Vero cells (Figure 6I). Upon overexpressing HA‐GADD45A and Flag‐V in HEK293T cells, we observed that GADD45A promotes the expression of the V protein in a dose‐dependent manner (Figure 6J). In endogenous experiments, transfecting Flag‐V into HEK293T cells demonstrated that V inhibits the expression of TBK1 protein in a dose‐dependent manner (Figure 6K). Furthermore, when Flag‐V was overexpressed in HEK293T cells and stimulated with Poly(I:C), we found that V significantly downregulated the expression of TBK1 (Figure 6L).

### 3.7 V and GADD45A Synergistically Promote PPRV Replication

**FIGURE 7:**
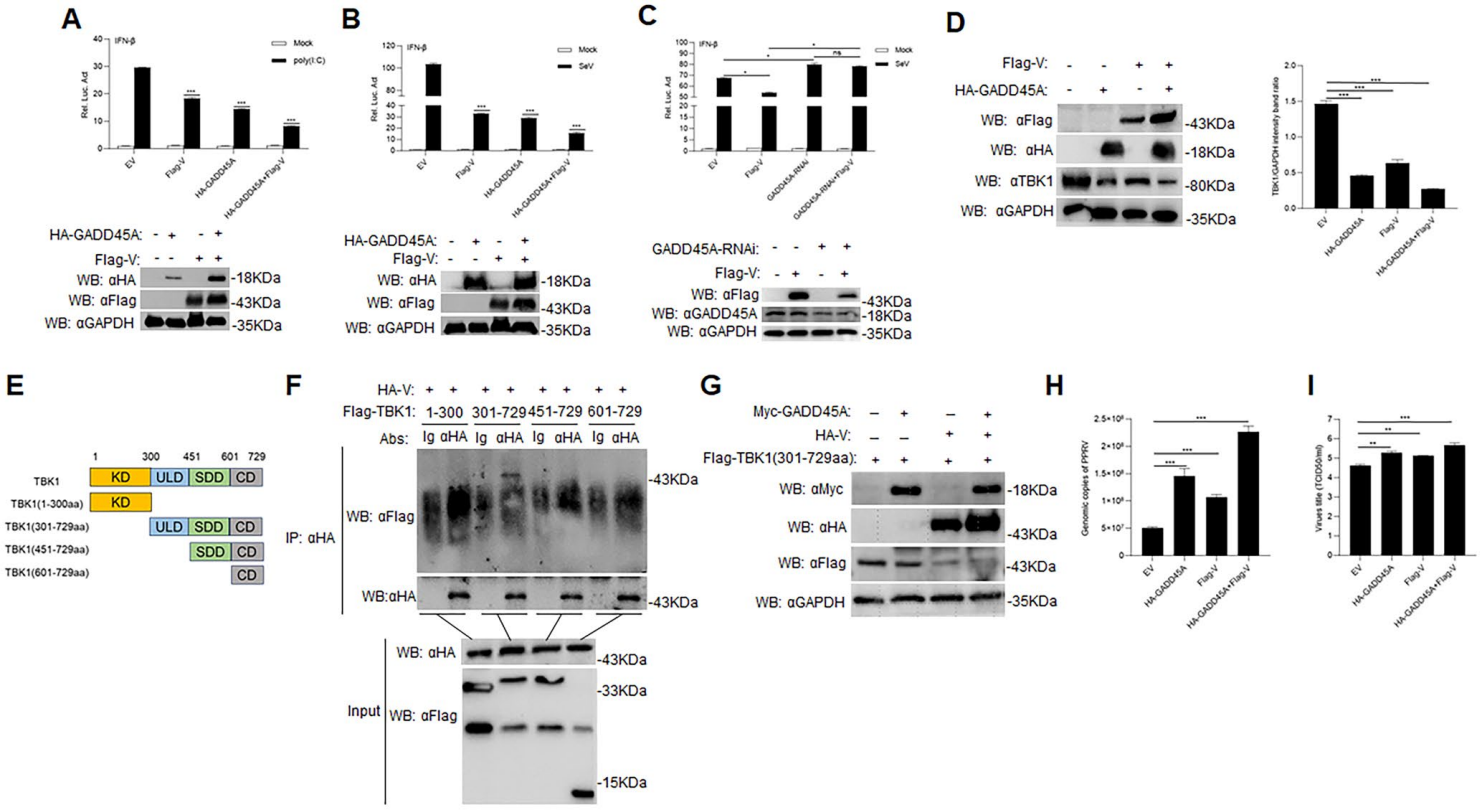
V and GADD45A synergistically promote PPRV replication. (A, B) The IFN‐β reporter gene (0.1 μg) was cotransfected with the indicated plasmids into HEK293T cells (1 × 10^5^) for 24 h. The cells were infected with SeV for 12 h or transfected with Poly(I:C) for 18 h. Luciferase assays and Western blot analyses were performed. (C) HEK293T cells were transfected with GADD45A‐specific RNAi for 48 h. Subsequently, the cells were co‐transfected with an IFN‐β luciferase reporter plasmid (0.1 μg) and the indicated plasmids for 24 h. Following transfection, cells were either infected with Sendai virus (SeV) for 12 h or left untreated. Luciferase activity was then measured, and cell lysates were collected for Western blot analysis. (D) Flag‐V and HA‐GADD45A plasmids were transfected individually or co‐transfected into HEK293T cells (2 × 10^5^) in 12‐well plates for 24 h. Empty vector (EV) transfection was used as a negative control. Samples were collected for Western blot analysis, quantification of TBK1 protein levels normalized to GAPDH by densitometry. (E, F) HA‐V (10 μg) was co‐transfected with Flag‐TBK1 (1‐300aa), Flag‐TBK1 (301‐729aa), Flag‐TBK1 (451‐729aa), or Flag‐TBK1 (601‐729aa) (10 μg) into HEK293T cells (2 × 10^6^) in 10‐cm dishes for 24 h. Co‐IP was performed using an anti‐HA antibody, followed by Western blot analysis. (G) Flag‐TBK1 (301‐729aa) was transfected alone or co‐transfected with Myc‐GADD45A or HA‐V into HEK293T cells (2 × 10^5^) in 12‐well plates for 24 h. Samples were collected and subjected to Western blot analysis using specific antibodies. (H, I) Flag‐V and HA‐GADD45A plasmids were transfected individually or co‐transfected into Vero cells (2 × 10^5^) for 24 h. The cells were then infected with PPRV (MOI = 1.0) for 24 h. The PPRV genome copy number (G) and viral titer (H) were measured. Data are presented as the mean ± SD (*n* = 3). *, *p* < 0.05; **, *p* < 0.01; ***, *p* < 0.001.

To further explore the roles of GADD45A and V in PPRV replication, we transfected HEK293T cells with Flag‐V, HA‐GADD45A for 24 h, followed by transfection with Poly(I:C) or SeV. The results demonstrated that both V and GADD45A reduce the activation of the IFN promoter (Figure 7A,B; *p* < 0.001). To further investigate the role of GADD45A in V protein–mediated inhibition of interferon signaling, we performed RNA interference (RNAi) experiments using small interfering RNAs specifically targeting GADD45A. Consistent with our previous findings, expression of the V protein significantly suppressed IFN‐β activation in wild‐type cells. However, upon depletion of GADD45A, IFN‐β expression was markedly upregulated, and the ability of the V protein to inhibit IFN‐β induction was largely abrogated. Notably, we also observed a reduction in V protein expression levels in cells with GADD45A knockdown (Figure 7C). Moreover, endogenous experiments indicated that both V and GADD45A inhibit TBK1 expression, and their combined effects further suppressed TBK1 protein levels (Figure 7D; *p* < 0.001). By constructing TBK1 truncations (Figure 7E), we identified that V interacts specifically with the TBK1 (301‐729aa) region (Figure 7F). When Myc‐GADD45A, HA‐V, and Flag‐TBK1 (301‐729aa) were co‐transfected into HEK293T cells, the results revealed that both GADD45A and V inhibit the expression of Flag‐TBK1 (301‐729aa), with a synergistic suppression observed (Figure 7G). We transfected Vero cells with HA‐GADD45A and Flag‐V either individually or together for 24 h, followed by infection with PPRV. PPRV replication was significantly increased in Vero cells overexpressing V or GADD45A alone compared to the Mock group; furthermore, PPRV replication was even more pronounced in Vero cells co‐expressing V and GADD45A (Figure 7H,I; *p* < 0.01).

## 4 Discussion

GADD45A plays diverse roles in cellular processes, including DNA damage repair, cell cycle regulation, modulation of signaling pathways, antiviral responses, stress responses, and cell differentiation and development [28]. Following viral infection, the interactions between viral proteins and host proteins are pivotal for successful viral replication and dissemination. Notably, GADD45A is upregulated in Hepatitis B virus X (HBx) protein‐induced carcinogenesis [26]. In this study, we observed a significant increase in GADD45A transcription levels correlating with both the time and dose of PPRV infection. The GADD45A facilitated viral replication, whereas GADD45A knockdown resulted in a marked reduction in viral replication (Figure 1).

Virus infection activates receptors such as RIG‐I, which leads to the recruitment of the adaptor protein MAVS to the RIG‐I‐like receptor (RLR) signaling complex. This process induces the expression of type I interferons (IFN‐I) and other antiviral genes, thereby establishing an antiviral state [29]. Research has shown that Zinc Finger NFX1‐Type Containing 1 (ZNFX1) interacts with MAVS and promotes the expression of IFN and ISGs, effectively restricting RNA virus replication [30]. The accessory protein Vpr of human immunodeficiency virus type‐1 (HIV‐1) induces apoptosis by activating ATR, which leads to BRCA1 phosphorylation and subsequently upregulates GADD45A [31]. In transgenic mice with skeletal muscle‐specific expression of GADD45A, it has been observed that GADD45A induces a reduction in skeletal muscle mitochondria and oxidative capacity, selectively causing atrophy of glycolytic muscle fibers, while paradoxical promoting expression of oxidative myosin heavy chain despite the loss of mitochondrial loss [32]. In this study (Figures 3, 4, 5), we found that the overexpression of GADD45A significantly downregulated the transcription of ISGs factors. We confirmed that GADD45A interacts with TBK1. In TBK1‐knockout cells, the transcription of IFN‐β and ISG56 was markedly inhibited. GADD45A was shown to inhibit TBK1 expression in a dose‐dependent manner; following PPRV infection, GADD45A significantly suppressed TBK1 protein levels, when GADD45A was knocked down, TBK1 expression was notably higher than in the control group, and viral replication was inhibited. These findings suggest that GADD45A may act as a negative feedback regulator, inhibiting TBK1 activity to prevent excessive immune responses.

Viruses need to engage in various interactions with host proteins to effectively enter host cells and replicate within them. These interactions are crucial for different stages of the viral life cycle and play a significant role in helping viruses evade detection and elimination by the immune system. The A137R protein of African swine fever virus (ASFV) targets TBK1 to inhibit autophagy‐mediated lysosomal degradation, thereby negatively regulating the cGAS‐STING‐mediated IFN‐β signaling pathway [33]. Porcine epidemic diarrhea virus (PEDV) non‐structural protein 2 diminishes innate antiviral immunity by targeting TBK1 for NBR1‐mediated selective autophagy [34]. Glucose‐regulated protein 78 (GRP78) interacts with the envelope (E) protein of Zika virus (ZIKV), facilitating the internalization of ZIKV into cells, which is critical for effective replication [35]. Nonstructural protein (NSs) of Severe Fever with Thrombocytopenia Syndrome Virus (SFTSV) sequesters antiviral proteins such as TBK1 into autophagic vesicles, thereby promoting the degradation of TBK1 and other antiviral proteins [36]. Encephalomyocarditis virus (EMCV) protein 2C interacts with MDA5, which inhibits the activation of the IFN‐β signaling pathway [37]. Here, we have confirmed that GADD45A interacts with the V protein of PPRV and that the V protein interacts with TBK1, leading to the inhibition of TBK1 expression. Besides, overexpression of the exogenous V protein leads to a reduction in TBK1 expression. Notably, following poly(I:C) stimulation, the downregulation of TBK1 is significantly more pronounced in cells overexpressing the V protein compared to those treated with poly(I:C) alone. This finding suggests that the PPRV V protein can interfere with host antiviral signaling pathways, particularly the TBK1‐dependent pathway, which plays a central role in the activation of the type I interferon response. These results indicate that the V protein may specifically target key components of the innate immune response, potentially by inhibiting TBK1 activation or destabilizing its protein structure (Figure 6). Both GADD45A and the V protein can work synergistically to inhibit the activation of the IFN‐β promoter and suppress TBK1 expression, thereby promoting PPRV growth. The PPRV V protein can evade host immunity by interacting with the host protein GADD45A, which inhibits TBK1 expression and facilitates viral replication in the host (Figure 7). This study suggests that during PPRV infection, GADD45A inhibits TBK1, possibly via its interaction with the V protein of PPRV, leading to a synergistic effect, thus promoting PPR replication, effectively suppressing the host's antiviral immune response, thereby promoting viral replication and dissemination.

The Morbillivirus genus includes Rinderpest virus (RPV), PPRV, Measles virus (MeV), and Canine distemper virus (CDV). The non‐structural V proteins of those viruses could effectively block the activity of type I IFN [38]. Our research has demonstrated that the PPRV V protein interacts with the host protein GADD45A, leading to the downregulation of TBK1 expression and a cooperative inhibition of the activation of the IFN‐β promoter, which in turn promotes the replication of PPRV in host cells. This indicates that the V protein of PPRV can bind to host proteins, suppress interferon‐mediated antiviral gene expression, disrupt innate immune signaling pathways, and enhance the virus's infectivity and pathogenicity.

**FIGURE 8:**
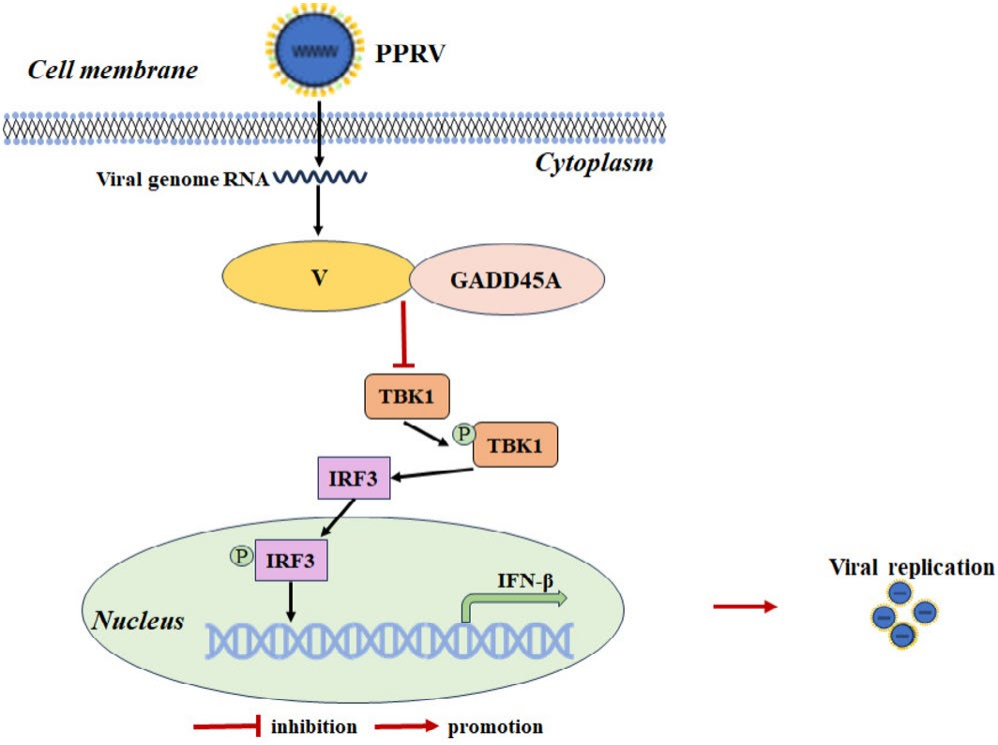
A working model of host protein GADD45A regulation of PPRV replication. During PPRV infection, the viral protein V forms a complex with GADD45A in the cytoplasm. GADD45A interacts with TBK1 and inhibits its expression. Similarly, V also interacts with TBK1 and inhibits TBK1 expression. The synergistic inhibition of TBK1 by both GADD45A and V leads to the downregulation of IFN‐β promoter activation, ultimately promoting PPRV replication.

Based on these findings, we propose a working model (Figure 8) in which GADD45Amediates the promotion of PPRV replication. During PPRV infection, the viral protein V forms a complex with GADD45A. The synergistic inhibition of TBK1 by both GADD45A and V ultimately leads to the downregulation of the activation of the IFN‐β promoter, negatively regulating cellular antiviral responses, thereby promoting PPRV replication. These findings provide a theoretical basis for further exploration of the interactions between PPRV and host proteins.

## Author Contributions

**Dan Li, Youjun Shang and Haixue Zheng:** conceptualization. **Haiyan Ding, Wenping Yang, Jinyan Wu, Junhuang Wu, and Mengyi Wang:** methodology. **Jijun He, Ligang Yuan, Jinyan Wu, Wenping Yang, and Haixue Zheng:** formal analysis. **Dan Li, Haiyan Ding, and Youjun Shang:** investigation. **Dan Li, Youjun Shang, and Haiyan Ding:** writing of the original draft. **Dan Li, Youjun Shang, and Haiyan Ding:** review and editing of the manuscript. **Dan Li and Youjun Shang:** supervision. **Youjun Shang and Jijun He:** funding acquisition.

## Conflicts of Interest

The authors declare no conflicts of interest.

## Data Availability

All data supporting the findings of this study are available within this paper.
